# Usability and Acceptance Testing of an Electronic Patient-Reported Outcome Symptom Monitoring System for People Receiving Immune Checkpoint Inhibitors: Mixed Methods Study

**DOI:** 10.2196/79694

**Published:** 2026-03-18

**Authors:** Julia Lai-Kwon, Claudia Rutherford, Stephanie Best, Hasan Shahid Ferdous, Iris Zhang, Thai-Khoa Ly, Dishan Herath, Kate Burbury, Michael Jefford

**Affiliations:** 1 Department of Medical Oncology, Peter MacCallum Cancer Centre, Melbourne, Victoria, Australia; 2 Department of Health Services Research, Peter MacCallum Cancer Centre, 305 Grattan St, Melbourne, Victoria, 3000, Australia, 61 385595000; 3 Sydney Quality of Life Office, Susan Wakil School of Nursing and Midwifery, Faculty of Medicine and Health, University of Sydney, Sydney, New South Wales, Australia; 4 Melbourne School of Health Sciences, University of Melbourne, Melbourne, Victoria, Australia; 5 Australian Genomics, Murdoch Children’s Research Institute, Melbourne, Victoria, Australia; 6 Centre for Digital Transformation of Health, University of Melbourne, Melbourne, Victoria, Australia; 7 Peter MacCallum Cancer Centre, Melbourne, Victoria, Australia; 8 Sir Peter MacCallum Department of Oncology, University of Melbourne, Melbourne, Victoria, Australia; 9 Australian Cancer Survivorship Centre, Peter MacCallum Cancer Centre, Melbourne, Victoria, Australia

**Keywords:** symptom monitoring, patient-reported outcomes, electronic patient-reported outcomes, digital health, usability, acceptance, immunotherapy, immune checkpoint inhibitors

## Abstract

**Background:**

Immune checkpoint inhibitors are widely used in oncology but can cause immune-related adverse events (irAEs), which may be severe or life-threatening if not detected early. Electronic patient-reported outcome (ePRO) symptom monitoring systems may facilitate timely recognition and management of irAEs. Usability testing is a critical stage in ePRO system development, yet no published examples of formal usability and acceptance testing exist.

**Objective:**

This study aims to assess the usability and acceptance of a co-designed ePRO symptom monitoring prototype for irAEs embedded within the Epic electronic medical record.

**Methods:**

Testing was conducted at an Australian quaternary cancer center. Eligible participants were patients who had received or were receiving immune checkpoint inhibitors, their caregivers, or clinicians (oncologists and nurse specialists). Participants completed baseline digital literacy assessments (16-item Mobile Device Proficiency Questionnaire [MDPQ-16] and 12-item Computer Proficiency Questionnaire [CPQ-12]) before a structured testing session. Each session involved role-specific tasks using the patient-facing Health Hub or the clinician-facing Epic electronic medical record. Usability was assessed using the System Usability Scale (SUS). Acceptance was assessed using a customized Unified Theory of Acceptance and Use of Technology (UTAUT) questionnaire. Semistructured interviews were used to capture qualitative feedback.

**Results:**

A total of 30 participants (7 patients, 3 caregivers, 10 oncologists, and 10 nurse specialists) completed 10 testing sessions. Median MDPQ-16 and CPQ-12 scores were higher for clinicians compared to patients and caregivers. Median SUS scores indicated high usability—patients and caregivers: 77.5% (IQR 70.0%‐86.3%), oncologists: 82.5% (IQR 80.0%‐90.0%), and nurse specialists: 80.0% (IQR 75.6%‐94.4%). Median UTAUT scores demonstrated strong user acceptance—patients or caregivers: 4.27 (IQR 4.09‐4.58), oncologists: 4.33 (IQR 4‐4.63), and nurse specialists: 4.23 (IQR 3.87‐4.57). Health Hub usability themes highlighted overall ease of navigation and efficiency of reporting, but a need for clearer survey navigation, simplification of the actions page, and improved organization of trend graphs. For clinicians, themes included efficient side effect capture and intuitive system design, but a need to improve navigation to results, optimize data display, and facilitate team-based alert management. Health Hub acceptance themes highlighted patient empowerment to self-manage, enhanced patient-clinician communication, and reinforcement of existing care. However, concerns were raised about digital equity for vulnerable groups. Clinicians reported that the system streamlined side effect management between visits, aligned with existing Epic workflows, and could be tailored to personal preferences. Concerns remained regarding additional workload and medico-legal responsibilities associated with real-time alerts.

**Conclusions:**

The ePRO prototype demonstrated high levels of usability and acceptance across patients, caregivers, and clinicians. Limitations around navigation and data visualization, alongside equity and workload concerns, will guide refinements prior to implementation. These findings emphasize the value of rigorous formative usability and acceptance testing to optimize ePRO systems prior to deployment in routine cancer care.

## Introduction

Immune checkpoint inhibitors (ICI), which block inhibitory signals of T-cell activation to promote antitumor immune responses, are now a standard of care for people with various cancer types. While ICIs have significantly improved clinical outcomes, they are also associated with immune-related adverse events (irAEs). These events can present with a variety of symptoms [1], may be severe or life-threatening [2-5], and are associated with increased acute health care use and health care costs [6-9]. Common irAEs involve organs such as the skin (eg, rash and pruritus) and the gastrointestinal tract (eg, diarrhea) [10].

Between clinic visits, people receiving ICIs and their caregivers are primarily responsible for identifying and reporting symptoms suggestive of an irAE. However, recognizing irAEs can be challenging and a source of anxiety for patients and caregivers [11-13]. Digital tools, such as electronic patient-reported outcome (ePRO) symptom monitoring, may support people to self-monitor for irAEs and facilitate early reporting of concerning symptoms to health care professionals. There is high-quality evidence demonstrating the benefits of ePRO symptom monitoring at the patient, clinician, and health care system level [14,15].

We co-designed a real-time ePRO symptom monitoring system for people receiving ICI in collaboration with end users [16]. The prototype was first developed using eSyM (Epic Systems Corporation), an ePRO symptom management program that is integrated into the Epic electronic medical record (EMR) system [17,18], as the foundation. We then adapted this prototype to address the specific needs of people receiving ICI and to align with local preferences and workflows. The final prototype consisted of a patient-facing smartphone app and website known locally as “Health Hub” (MyChart; Epic Systems Corporation) and the clinician-facing Epic EMR.

Usability testing is a critical stage in the development of digital health care tools [19]. The International Organization for Standardization defines usability as the “extent to which a product can be used by specified users to achieve specified goals with effectiveness, efficiency, and satisfaction in a specified context of use” [20]. Usability testing is the “formal assessment of the extent to which a product or system is effective, efficient, and perceived as satisfactory by users” [19]. For ePRO systems, testing would involve end users performing predetermined tasks and providing feedback. This ensures the intended purpose of the ePRO system is achieved and that users are satisfied with their user experience.

High levels of usability and acceptance are critical for ensuring user engagement and long-term sustainability of ePRO symptom monitoring. Performing usability testing iteratively during a system’s development will ensure that any issues are detected and addressed prior to implementation, ensuring the final product is fit for purpose, which may help to reduce attrition rates postimplementation [19]. However, there are no published examples of formal usability testing of ePRO systems.

The usability and acceptance of our ePRO system have not been established, and formal usability and acceptance testing of the eSyM system has not been published. Guided by the principles described by Aiyegbusi [19] for usability testing of ePRO systems, this study aimed to assess the usability and acceptance of our ePRO system with end users (patients, caregivers, and clinicians).

## Methods

### Study Population

Patient participants were older than 18 years and were receiving or had received ICI. Caregiver participants were older than 18 years and had direct caring responsibility for a person who was receiving or had received ICI. Clinician participants were oncologists and nurse specialists involved in caring for people receiving ICI.

Participants were identified from co-design workshops undertaken as part of preliminary work to develop the ePRO system [16] and contacted directly to discuss this study. This enabled people to participate across the entire system co-design process. Additional patient and caregiver participants were identified by screening melanoma and lung clinic lists and approached via their treating physician. These clinics were chosen as many patients and caregivers had prior ICI experience. A research assistant (IZ) then called the patient and caregiver to discuss the study and arrange consent. Additional clinician participants were identified via the research team’s professional networks and were emailed the consent form.

Sample size was determined by a number of factors [19,21]. First, for iterative testing, prior studies recommend at least 5 participants per test cycle to detect 85% of issues [22]. Second, the degree of end-user heterogeneity (eg, age and digital experience) and potential dropout should be considered [23,24]. Third, the complexity of the physical and cognitive tasks should also be considered, with 5‐10 participants recommended for simple ePRO systems and up to 20 for more complex systems [25]. However, it is not clear whether this refers to each test cycle or the entire testing process [25]. Finally, the nature of the data collected should also be considered. For example, if data are predominantly qualitative rather than quantitative, sample size will be influenced by the achievement of thematic saturation [26]. To account for these factors, we aimed to recruit 10 patients or caregivers, 10 oncologists, and 10 nurse specialists to the first round of usability testing.

Our system was developed using Responsive Web Design, which enables dynamic adaptation to different screen sizes and orientations [27]. To account for the similarity between mobile and tablet interfaces [28], we aimed to test 5 patients or caregivers using a mobile phone or tablet and 5 patients or caregivers using a computer.

### Data Collection

After completing the participant information and consent form, participants completed 2 online questionnaires via REDCap (Research Electronic Data Capture): the 16-item Mobile Device Proficiency Questionnaire (MDPQ-16) [29] and the 12-item Computer Proficiency Questionnaire (CPQ-12) [30] to assess their digital literacy. The MDPQ-16 assesses participants’ ability to use mobile devices with each item measured on a 5-point scale (“never tried” to “very easily”). The MDPQ-16 has demonstrated excellent reliability (Cronbach *α*=0.96) [29]. The CPQ-12 assesses participants’ computer proficiency with each item measured on a 5-point scale (“never tried” to “very easily”). The CPQ-12 has demonstrated excellent reliability (Cronbach *α*=0.98) [30]. For both questionnaires, higher scores indicate higher levels of proficiency. The MDPQ-16 and CPQ-12 were chosen because they were designed to assess digital literacy and are widely used. Participants were also provided with prereading materials outlining the aims of the testing session, session format, and anticipated outputs (Section 1 in Multimedia Appendix 1).

**Figure 1.**
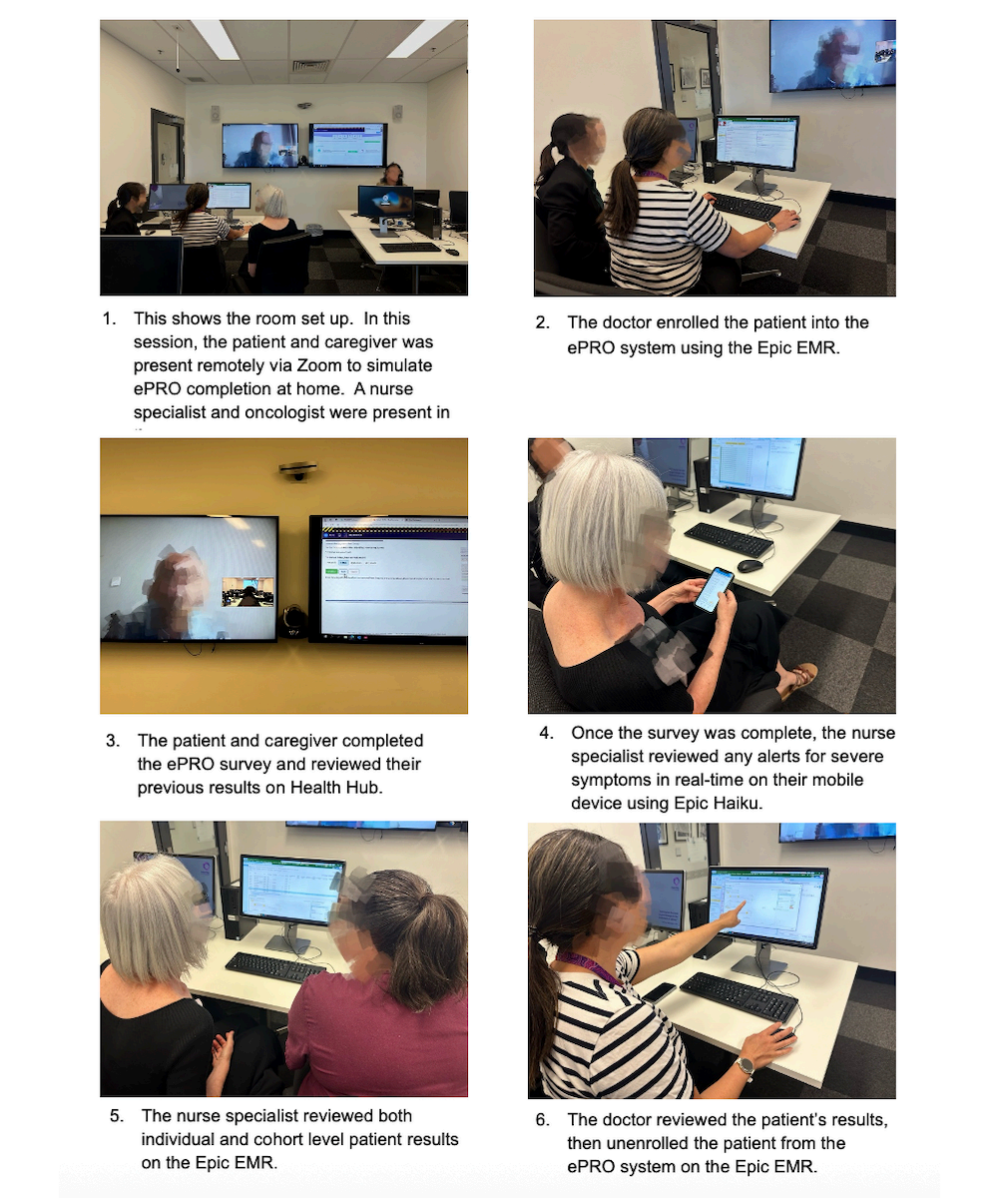
Room setup for usability and acceptance testing. EMR: electronic medical record; ePRO: electronic patient-reported outcome.

The usability and acceptance testing sessions were cofacilitated by JLK (a medical oncologist) and IZ (a research assistant). Each session lasted 1.5 hours. Seven sessions were conducted with all participants on-site, and 3 were conducted with the patient and caregiver present remotely via Zoom (Zoom Communications, Inc) to simulate ePRO completion at home. Figure 1 demonstrates the setup of the testing room. All individuals shown provided consent for their images to be included in this paper. All participants were briefed that the purpose of the session was to test the ePRO system interface, not assess the meaning or clinical relevance of questions within the ePRO survey [19].

**Figure 2.**
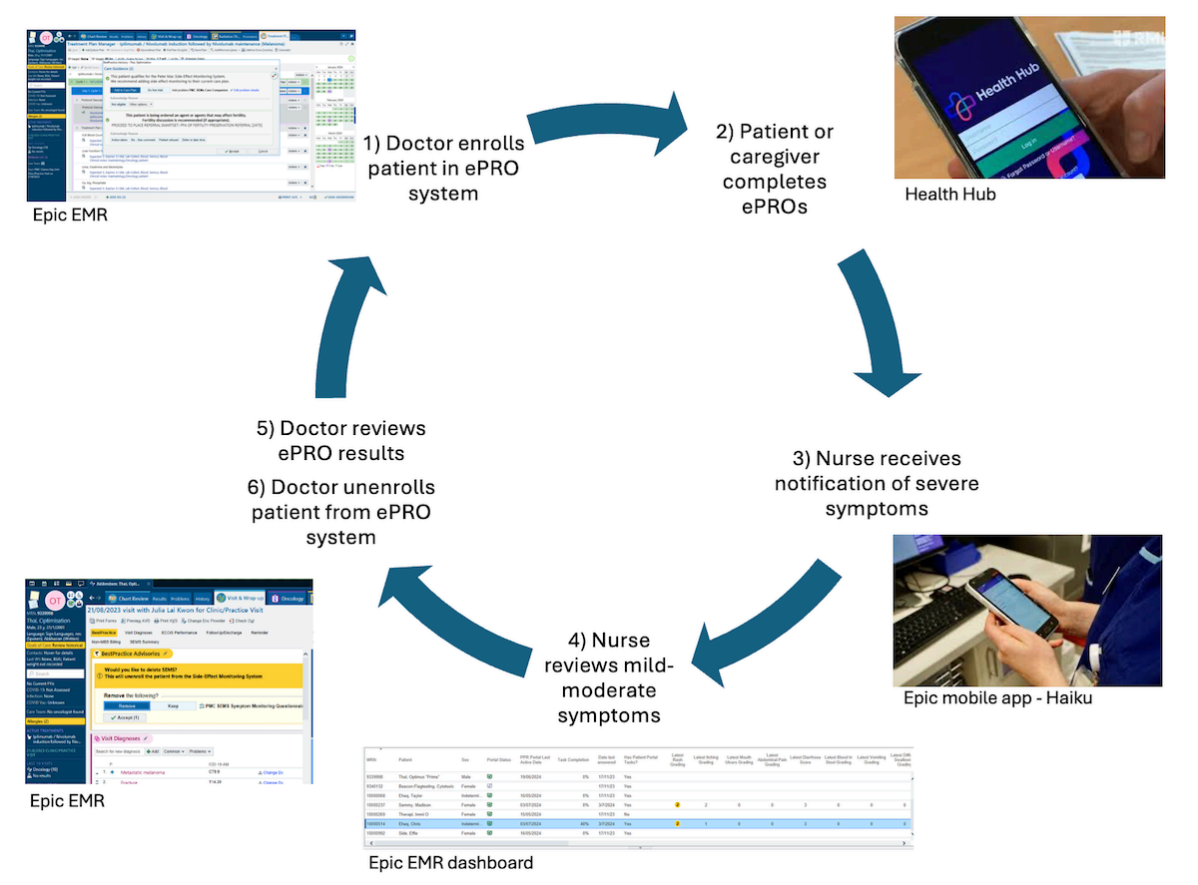
Usability and acceptance testing procedure. EMR: electronic medical record; ePRO: electronic patient-reported outcome.

During each session, participants were first trained on a series of tasks that reflected their role (Section 2 in Multimedia Appendix 1), then performed those tasks independently while being observed by JLK and IZ. Patients and caregivers tested the Health Hub and clinicians tested the Epic EMR. Testing was performed as a group to reflect how the ePRO system would function in real-world practice, with the doctor enrolling the patient and caregiver into the system, the patient and caregiver completing the survey and reviewing their previous results in Health Hub, the nurse specialist reviewing alerts for severe symptoms in real-time and individual- and cohort-level patient results, and the doctor reviewing results and unenrolling the patient (Figure 2). Participant actions, verbal, and nonverbal cues were noted by JLK and IZ for probing during the qualitative interview.

Participants then completed a qualitative, semistructured interview regarding their experience using the system using the retrospective probing technique [19]. The interview schedule is shown in Section 2 in Multimedia Appendix 1.

Interviews were audio-recorded and transcribed using the automated transcription feature on Microsoft Teams. Transcripts were checked by IZ (a research assistant) against audio recordings to ensure their accuracy and deidentify. Participants were not invited to review their transcripts but consented to being contacted if further clarification of their comments was required. Participants were reimbursed with an AUD $50 voucher (US $33.41) for their attendance in accordance with the hospital’s consumer engagement policy.

Following the testing session, participants completed 2 questionnaires on REDCap: the System Usability Scale (SUS) [31] and a customized questionnaire based on the Unified Theory of Acceptance and Use of Technology (UTAUT) [32,33] (Section 3 in Multimedia Appendix 1). The 10-item SUS evaluates the usability of software, mobile devices, websites, and apps (ie, the extent to which a system can be used effectively, efficiently, and satisfactorily) using 5 response options (“strongly agree” to “strongly disagree”). In contrast, the 15-item UTAUT assesses the degree of user acceptance of digital systems, capturing user perceptions about performance expectancy, effort expectancy, social influence, and facilitating conditions using a 5-point Likert scale (“strongly disagree” to “strongly agree”). Together, these tools provide complementary insights, with the SUS quantifying participants’ direct interactions with the system to assess whether the system is easy to use, while the UTAUT assesses the likelihood of sustained engagement and integration into routine workflows. For both questionnaires, higher scores indicated higher levels of usability or acceptance. These questionnaires were chosen as they assessed the desired outcomes (usability and acceptance) and were widely used for digital health interventions.

### Data Analysis

Questionnaire data regarding digital literacy, system usability, and user acceptance were analyzed in RStudio (version 4.4.1; Posit PBC). Descriptive statistics were used to summarize responses.

Content analysis [34] was used to identify responses regarding the usability and acceptance of the prototype ePRO monitoring system. Transcripts were deidentified and read line by line by JLK and IZ to familiarize themselves with the dataset. To ensure coding rigor, 20% of transcripts were coded by both JLK and IZ. Once codes were agreed upon by both JLK and IZ, the remaining transcripts were coded by IZ. Transcripts were coded in NVivo (version 12; Lumivero). Codes were grouped into themes and subthemes relating to the usability and acceptance of the system identified by the study participants. Any disagreements were resolved by discussion with coauthors. All researchers were involved in reviewing the themes and subthemes, and final interpretations were arrived at by consensus. Saturation was defined as no new usability or acceptance concepts being identified for each of the 3 participant groups (patients and caregivers, oncologists, and nurse specialists) and confirmed jointly by JLK and IZ.

### Ethical Considerations

We conducted usability and acceptance testing sessions at a quaternary cancer center in Australia. Institutional ethics approval was granted by the Peter MacCallum Cancer Centre Low and Negligible Risk Research Ethical Review Committee (HREC/96744/Peter MacCallum Cancer Centre, August 17, 2023). Study participants were informed about the confidentiality of the data they provided, and written informed consent was acquired from all participants before being included in the study. Participants received an AUD $50 (US $33.41) voucher if they participated in a testing session. No additional incentives were offered for completing the presession and postsession questionnaires. All data collected were deidentified and stored on a secure server. The study was conducted in accordance with the National Statement on Ethical Conduct in Human Research and the Declaration of Helsinki. The authors confirm that the human research participants provided informed consent for publication of the images shown in Figure 1.

## Results

### Participant Characteristics

Thirty participants were recruited: 7 patients, 3 caregivers, 10 oncologists, and 10 nurse specialists (Tables1  2). Patients and caregivers were mostly male (7/10, 70%), aged 61‐70 years (5/10, 50%), spoke English at home (9/10, 90%), had an undergraduate degree (5/10, 50%), had melanoma or were caring for someone with melanoma (9/10, 90%), and had been receiving (or cared for someone receiving) ICI for 6‐12 months (5/10, 50%). All patients and caregivers had access to the Health Hub (10/10, 100%). Patients and caregivers were “somewhat confident” (3/10, 30%) or “quite confident” (3/10, 30%) using computers, smartphones, or other electronic devices. Median MDPQ-16 and CPQ-12 scores for patients and caregivers were 32.0 (IQR 22.5‐37) and 24.3 (IQR 22.5‐26), respectively.

**Table 1.** Patient and caregiver characteristics.

Characteristics	Total (n=10)
Age (years), n (**%**)	
41‐50	1 (10)
51‐60	1 (10)
61‐70	5 (50)
>70	3 (30)
Sex, n (**%**)	
Male	7 (70)
Country of birth, n (**%**)	
Australia	9 (90)
Other	1 (10)
Language spoken at home, n (**%**)	
English	9 (90)
Other	1 (10)
Role, n (**%**)	
Patient	7 (70)
Cancer type, n (**%**)	
Melanoma	9 (90)
Lung	1 (10)
Duration of immunotherapy treatment (months), n (**%**)	
6‐12	5 (50)
13‐18	1 (10)
>18	4 (40)
Access to Health Hub^a^, n (**%**)	
Yes	10 (100)
Easy to ask doctors questions regarding their health, n (**%**)	
Strongly agree	8 (80)
Slightly agree	2 (20)
Easy to ask nurses questions regarding their health, n (**%**)	
Strongly agree	7 (70)
Slightly agree	3 (30)
Confident in working with doctors to manage immunotherapy side effects, n (**%**)	
Very confident	5 (50)
Quite confident	4 (40)
Somewhat confident	1 (10)
Use of electronic devices^b^, n	
Smartphone	8 (80)
Tablet	6 (60)
Desktop or laptop	9 (90)
Self-reported confidence in using electronic devices for online activities, n (**%**)	
Very confident	2 (20)
Quite confident	3 (30)
Somewhat confident	3 (30)
A little confident	1 (10)
Not at all confident	1 (10)

a Health Hub: a secure, patient-facing mobile app and website linked with the electronic medical record, which provides information regarding appointments, medications, and some test results.

b Some participants provided multiple answers.

**Table 2.** Clinician characteristics.

Clinician characteristics	Total (n=20)
Age (years), n (**%**)	
18‐30	1 (5)
31‐40	6 (30)
41‐50	9 (45)
51‐60	2 (10)
61‐70	2 (10)
Sex, n (**%**)	
Male	5 (25)
Country of birth, n (**%**)	
Australia	12 (60)
Other	8 (40)
Language spoken at home, n (**%**)	
English	16 (80)
Other	4 (20)
Role, n (**%**)	
Oncologist	10 (50)
Nurse	10 (50)
Tumor stream, n^a^	
Melanoma and skin	8 (40)
Gynecological	6 (30)
Head and neck	5 (25)
Prostate	4 (20)
Bladder	4 (20)
Kidney	4 (20)
Lung	3 (15)
Breast	3 (15)
Gastrointestinal	3 (15)
Central nervous system	2 (10)
Sarcoma	2 (10)
Hematological	1 (5)
Colorectal	1 (5)
Pool (cover all streams)	1 (5)
Duration of experience in current role (years), n (%)	
<1	6 (30)
1‐4	3 (15)
5‐9	4 (20)
10‐14	6 (30)
>15	1 (5)

a Some participants provided multiple answers.

Out of 20 clinicians, oncologists and nurse specialists were mostly female (15/20, 75%), aged 41‐50 years (8/20, 45%), who had worked less than 1 year (6/20, 30%) or 10‐14 years (6/20, 30%) in their current role. The majority managed patients with melanoma and skin cancers (8/20, 40%) or gynecological cancers (6/20, 30%). Median MDPQ-16 scores for oncologists and nurse specialists were 39.0 and 39.0 (IQR 38.5‐40.0 and 36.6‐40.0, respectively) and median CPQ-12 scores were 29.0 and 29.8 (IQR 28.6‐30.0 and 28.2‐30.0, respectively).

### Usability and Acceptance Testing Sessions

Ten usability and acceptance testing sessions were conducted from July to October 2024. Each session consisted of 1 patient and caregiver, 1 oncologist, and 1 nurse specialist. All participants only completed 1 testing session. The median duration of each testing session was 70 (IQR 60.5‐79.5) minutes.

### Postsession Surveys

Median SUS scores for patients and caregivers, oncologists, and nurse specialists were 77.5%, 82.5%, and 80.0% (IQRs 70.0%‐86.3%, 80.0%‐90.0%, 75.6%‐94.4%), respectively. Median UTAUT scores for patients and caregivers, oncologists, and nurse specialists were 4.27, 4.33, and 4.23 (IQRs 4.08‐4.58, 4.00‐4.63, 3.87‐4.57, respectively).

### ePRO System Usability: Themes and Subthemes

Testing was continued until the point of qualitative data saturation, which was achieved after 10 interviews for each of the participant groups (patients and caregivers, oncologists, and nurse specialists).

Ten themes relating to ePRO system usability were identified (Table 3). Five themes related to the patient-facing Health Hub.

**Table 3.** Themes regarding usability of the electronic patient-reported outcome symptom monitoring system.

Digital Health Platforms and Themes	Illustrative quotes
Patient-facing Health Hub	
The ePRO^a^ system was easy to navigate	“Once I got into it, it was pretty...quick for me to get through.” (C1, male) “Fairly easy...as I got used to it, it’d be extremely easy (to navigate).” (C3, female)
The ePRO survey promoted efficient side effect reporting	“If you know what your side effects are...it can be quite fast. If you don’t have headaches or neuropathic pain...you just scroll past.” (P1, female) “Having it described as mild, moderate and severe...it's actually really great. Diarrhea is a good example...if I have loose, watery stools...a couple of times a week, is that moderate or is that severe? Should I contact the team?...I think the app helps to classify things...which I think...that's really useful.” (P6, male) “I loved the pictures...you can just see...that one's diarrhea and that one's itch.” (C3, female) “Maybe a sweet spot would be all images, but on a single page or even just something to note that there's one of five pages.” (P6, male)
Need for improved navigation within Health Hub	“I actually thought it was very easy for the first time...it's probably because I've been using (the) patient portal so regularly, that the format reminds me of it.” (P7, male) “Those initial first couple of menus, which was a bit like, ‘ok, what do I do here and where do I go’...that’s a little confusing.” (P6, male) “My one concern is...the trends thing...how important that is and…whether you’d remember to go back up to that menu icon.” (C1, male) “Yes...I think...you open it up, test results, visits, things like that. Something on that (home) page.” (P5, male)
Need for improved layout of the “immediate actions” page	“You could maybe simplify and just say phone patient nurse, self-monitor, here are some links.” (P6, male) “If it says you’ve got mild, then it should just say, ‘here’s the link’ and it shouldn’t go to the link…like, ‘here’s a library of fifty things’...it should be a hyperlink immediately to the one symptom that’s relevant.” (O3, female)
Need for improved layout of the “Trends Dashboard”	”It's good to have the record because it's hard to remember sometimes how bad something was and when it started...I think that's all good to have...the graphs and the records.” (P3, female) “I tend to forget what happened on what date. The nurse asks and I say I don’t know, it was a couple of weeks ago. But...if I've logged in or made a complaint or looked at something...with the dates...it’d help them a lot.” (P4, male) “I think when you’ve highlighted...what your own issues are, you might expect that it’d come...in order, but they’re in alphabetical order.” (P3, female) “If it’s in alphabetical order, you have to go load more, load more. If there was a button…you type rash, you look for the rash graph. It’s quicker.” (P2, male)
Clinician-facing Epic EMR^b^	
The ePRO system facilitated efficient collection of side effect data to improve patient care and quality improvement initiatives	“I really liked the concept. I think this is something that...as nurses have talked about how we can capture data about patients’ immunotherapy toxicities because we don’t see them that often...you rely on...their own reporting when they are...having their pre-treatment reviews.” (N9, female) “I think...from a data collection perspective and an inability to report what you do and justify why we struggle to keep up...will be helpful. (N1, female) “Hopefully we’ll see trends where we can have nurse-led interventions...this is where we thought we should be putting our efforts in but actually this is what we are missing.” (N3, female)
The ePRO system was easy to learn and intuitive to use	“It was very intuitive as to where to find things, so even though (I was) using it for the first time, I can sort of orientate myself and know which ones to click pretty easily.” (O4, male) “It was intuitive...if we didn't do the session, I think you would figure it out…relatively quickly anyway”. (O8, female) “I think there’s nothing really that makes this challenging from our end at all. I don’t think it adds workload.” (O6, male)
Need for improved navigation to patient ePRO results within Epic	“I couldn’t remember exactly where the results were...I guess that didn’t jump out at me…it blended in.” (N1, female) “If possible, have a separate tab on Epic...every time when you open it it’ll pop up as a kind of tab.” (N10, female) “...we have on the top right corner several small...icons that light up whenever there's...important results. But if there potentially could be something built that would have from a nurse's point of view…specific to this as symptoms reporter...that would...highlight up there. So when you're in a desktop, you can click that it will just pull up...symptoms which are quite severe and just list out the patients...of grade 3 and above. (O4, male)
Need for optimized display of individual- and cohort-level results	“I liked the...colour flags. I like that even when it is my first time seeing it on the phone. I could immediately go to severe.” (N3, female) “The ability to show the severity or the severe side effects very quickly with the colour coding...is really helpful.” (O1, female) “Having the ability to filter for a particular body system that you know generally gets a side effect and be able to push the rest to the bottom...if you...go, this one is notorious for GI but not for rash, you could filter it...could put GI at the top.” (N8, female) “What I didn't like particularly was just the amount of scrolling you had to do to get through all the symptoms. Would it be easier if there's something that comes up like just all the ones that are reported as an issue are the ones that come to the top.” (N2, female) “If you could have any indication of trend...that might add more information but not very much screen space.” (O6, male) “I think...have just a touch point that shows admission so that you know that you’re going to have an absence of data from that point...as opposed to not being able to appreciate the change in the trend based on a very significant event.” (O1, female)
Additional features to facilitate team-based alert management	“We have 3 and some other units have 5. And they are all working at a different time, different date...you don't want to have...people thinking someone else is doing it.” (N10, female) “It will require the nurses to have the training and knowledge to...make sure that we’re following up...the patients...there needs to be clear communication within our teams to make sure we are looking at the data, figuring out which teams need to see it.” (N6, female) “Have a tick box or something so they don’t have to see the notes, they can just go ‘it’s done.” (N5, female) “You need a way of flagging it in like a basket...that it has been addressed.” (O8, female)

a ePRO: electronic patient-reported outcome.

b EMR: electronic medical record.

#### The ePRO System Was Easy to Navigate

Patients and caregivers described the ePRO system as:

*fairly easy... it’d be extremely easy* (to navigate),

[C3, female]

even after a single training session.

#### The ePRO Survey Promoted Efficient Side Effect Reporting

Patients and caregivers felt that the ePRO survey facilitated efficient side effect reporting by using plain language, images to aid understanding of side effects:

*I loved the pictures…you can just see…diarrhea and ... itch,*

[C3, female]

and the “traffic light” color system reflecting the severity of each side effect. Suggestions to improve the efficiency of side effect reporting included displaying all side effects on the first page to shorten the survey or displaying progress through the survey.

#### Need for Improved Navigation Within Health Hub

The ePRO survey was housed within the broader Health Hub. Many found navigating from the Health Hub home page to the ePRO survey challenging. Other confusing features of the Health Hub included navigating to the “Trends Dashboard”:

*my one concern is...the trends thing... and...whether you’d remember to go back up to that menu icon.*

[C1, male]

Suggestions included having an ePRO system icon on the home page to improve navigation to the ePRO survey, naming functions such as the “Trends Dashboard” more intuitively, and removing hyperlinks used to move between pages.

#### Need for Improved Layout of the “Immediate Actions” Page

The “immediate actions” page indicated what side effects a patient and caregiver had reported, how severe these side effects were, and provided instructions about what to do immediately about their reported side effects. Patients and caregivers felt this page could be optimized by simplifying the language:

*you could ...simplify and just say phone patient nurse, self-monitor, ...links.*

[P6, male]

Clinicians also felt this page could incorporate links to a graph of that person’s side effect over time and specific self-management advice, rather than having this a separate task to complete later.

#### Need for Improved Layout of the “Trends Dashboard”

Patients and caregivers found the “Trends Dashboard” helpful for reviewing past results and noting changes in side effects over time. However, the presentation of the graphs in alphabetical order made it difficult to find relevant side effects:

*I think when you’ve highlighted…what your own issues are, you might expect that it’d come…in order.*

[P3, female]

Suggestions for improving the usability of these graphs included allowing sorting or filtering of the graphs by newness or severity or enabling people to search for relevant graphs.

Five themes related to the clinician-facing Epic EMR interface:

#### The ePRO System Facilitated Efficient Collection of Side Effect Data to Improve Patient Care and Quality Improvement Initiatives

Clinicians felt that the ePRO system facilitated proactive, standardized, and systematic capture of side effect data from all patients. Nurses saw value in using aggregated data to inform the business case for additional staff:

*I think…from a data collection perspective …will be helpful*

[N1, female]

or quality improvement initiatives:

*hopefully we’ll see trends where we can have nurse-led interventions...*

[N3, female]

#### The ePRO System Was Easy to Learn and Intuitive to Use

Clinicians found the ePRO system intuitive to use because it was integrated within existing clinician workflows in the Epic EMR. As a result, oncologists felt that the impact on their workload would be minimal:

*I don’t think it adds workload.*

[O6, male]

#### Need for Improved Navigation to Patient Results Within Epic

While clinicians felt navigation within Epic was relatively straightforward, some found the EMR interface cluttered, making it difficult to identify where ePRO results were kept:

*I couldn’t remember exactly where the results were...it blended in.*

[N1, female]

Suggestions were made to create a separate “tab” for ePRO symptom monitoring results within the EMR. If this was not possible, then banners that link to an individual patient’s results should be made more prominent.

#### Need for Optimized Display of Individual- and Cohort-Level Results

The “traffic light” color coding of side effect severity was highly valued by both oncologists and nurse specialists for identifying severe side effects quickly. Suggestions to optimize the display of ePRO results included the ability to sort an individual’s side effects by newness, severity, or body system, adding treatment-related time points to graphs, and using arrows to highlight side effect trends in the cohort-level dashboards:

*if you could have any indication of trend...that might add more information...*

[O6, male]

#### Additional Features to Facilitate Team-Based Alert Management

Nurse specialists highlighted that symptom monitoring was often performed by teams of nurses, and that the ePRO system should facilitate team-based alert management:

*It will require the nurses to have the training and knowledge to…make sure that we’re following up...the patients...there needs to be clear communication within our teams...*

[N6, female]

Suggestions included adding an acknowledgment system for resolved alerts in the cohort-level dashboard or a system of sending severe alerts to a specific person or “in basket.”

### ePRO System User Acceptance: Themes and Subthemes

Eight themes relating to the user acceptance of the ePRO system were identified (Table 4). Four themes related to the patient-facing Health Hub:

**Table 4.** Themes regarding user acceptance of the electronic patient-reported outcome symptom monitoring system.

Digital Health Platforms and Themes	Illustrative quotes
Patient-facing Health Hub	
The ePRO^a^ system empowered people to self-manage their side effects	“You report the symptom...if it’s not severe...the library will tell you what to do and it’s a trusted source...otherwise you have to go to the internet...and sometimes...the advice is not applicable.” (P2, male) “I’ve had quite a few side effects in my journey...it’s really difficult to know what’s...important and...difficult to know when you should contact the care team...so having that classified I think is very useful.” (P6, male) “The whole list of fact sheets...I’m wondering whether the one that is relevant to them...comes to the top.” (N5, female)
The ePRO system enhanced patient-clinician communication about side effects	“It’s a good way to get across to your clinical nurse consultant...exactly where you are at.” (P5, male) “I felt like it was a bit hard to get to the nurse sometimes, and presumably if they're going to ring back if it's severe, that's really good to know.” (P3, female) “It’s a good way of capturing the people who are less likely to call us.” (N9, female) “People should have an understanding of when their submissions have been received, it’s been acted on...I’d find that helpful.” (C2, male)
ePRO symptom monitoring was a valuable extension to current care	“Being able to read the side effects...online, is really good...you can keep returning to that as often as you need to.” (P7, male) “When I first started on this journey...I had...been given reams and reams of paper information (which is)...just in the draw. With this system, you can just go there and have a look.” (P5, male) “Some people really like interacting with the technology...tracking their symptoms...this gives them an easy way to do it that’s build into something that they would already be using.” (N7, female)
Concerns regarding digital equity	“Some older, really sick people might find this a bit hard to deal with...maybe their specialist would not put them into this program.” (P3, female) “I think carers will be assisting, particularly if the patient hasn’t been feeling all that well...so the initial education session for both is important.” (C1, male) “I think there's ways around that...reporting maybe wouldn't be done at home, but it could be done before the consult...with an iPad or something that's maybe a bit bigger...I'm happy to help people do that.” (N9, female)
Clinician-facing Epic EMR^b^	
The ePRO system streamlined the process for responding to side effects between clinic visits	“...it’s identifying patients that you wouldn’t have necessarily followed up earlier. So definitely identifying the patients that you need to follow up is a good thing because there's patients that have symptoms that aren't contacting us.” (N6, female) “I like the concept a lot, something that will flag...this is...doing the work for us and telling us who we need to call, and for the others that are doing well, they potentially don’t need anything.” (N9, female) “I like the ability to run a report once a day...as a pool person, it gives you the ability to...get a quick summary of everyone...and what you need to do for the day.” (N7, female) “If clinicians haven’t got a system where they’re checking it all the time...the system...tells them (the patients) to call, so it’s a double up, which is good.” (N6, female) “It gives some good information that maybe we’re not cold calling someone...you’ve got some information beforehand to go ‘I’ve received this…we’re going to have a chat about this.” (N5, female)
Excellent alignment of ePRO system with existing Epic workflows	“I liked how there were multiple ways of doing things...like the treatment plan signing...no one forces you to enroll, but to consider it. If it wasn’t there, you’d forget about it.” (O2, female) “We may not be using ‘reports’ as a usual thing, so that...may be a new step.” (N2, female) “Having not used this system at all on my phone, it’s easier to find things on the Epic screen because I use it several times a day but I don't use my phone with the Epic app at all.” (N2, female)
The ePRO system was adaptable to each clinician’s personal workflow	“It was integrated pretty well into my normal workflow...it didn’t require me to go into any additional section to find exactly where it was.” (O4, male) “I think the nursing side is pretty easy too. Once you get into it, you’ll choose which way is easiest for you, if it’s open up the application first or...go straight to your report.” (N5, female) “I liked how there were multiple ways of doing things...like the treatment plan signing...no one forces you to enroll, but to consider it. If it wasn’t there, you’d forget about it.” (O2, female) “Whether they have access to those notifications or to be able to run those reports on a team...there should be a way within Epic to be able to...allocate them access to it.” (N5, female) “You may not get a reminder in a timely manner on your phone...even when I’m there on my computer, I’m not checking my phone or the Haiku app (Epic Systems Corporation) constantly...a chart alert thing would be good.” (O2, female)
Concerns regarding increased workload due to ePRO symptom monitoring	“I’m wondering if it’s open to over-reporting...they just want to be engaged and that could...become more of a hindrance than a help.” (P7, male) “Whenever you give someone power to report their problems...it really is variable on how much people are going to use it...if people report symptoms very...aggressively...which they might feel is a very valid thing to do...I can imagine that overwhelming people quite easily.” (O6, male) “I can see if they are under-reporting, which is always an issue even now...they go ‘it's a little bit.’ But when you talk to them...oh no, it's 10 times a day I go to the toilet.” (O2, female) “You could see potentially medico-legally, someone reported a severe grade 3...you just signed off and you didn’t see it and then you will be legally liable, you didn’t respond to the symptoms.” (O9, male) “Would you be able to add...on health hub that...please do not do this after hours?” (N1, female)

a ePRO: electronic patient-reported outcome.

b EMR: electronic medical record.

#### The ePRO System Empowered People to Self-Manage Their Side Effects

The ePRO system provided reassurance about how to immediately respond to side effects and was perceived as a trusted source of self-management advice:

*if it’s not severe...the library will tell you what to do and it’s a trusted source...otherwise you have to go to the internet...and sometimes...the advice is not applicable.*

[P2, male]

Improvement could include prioritizing the presentation of self-management advice in the education library to ensure people can find the relevant advice quickly:

*the one that is relevant to them...comes to the top.*

[N5, female]

#### The ePRO System Enhanced Patient-Clinician Communication About Side Effects

The ePRO system enhanced patient-clinician communication by providing clear instructions to patients about when and how to report side effects:

*It’s a good way to get across to your clinical nurse consultant…exactly where you are at*

[P5, male]

and enabling clinicians to monitor patient symptoms between visits to hospital, particularly

*the people who are less likely to call us.*

[N9, female]

Improvements could include an acknowledgment of ePRO status (ie, whether the survey had been received, reviewed, or actioned by the health care team).

#### ePRO Symptom Monitoring Was a Valuable Extension to Current Care

Participants found the ePRO system complemented and reinforced existing patient education about side effects:

*Being able to read the side effects...online, is really good...you can keep returning to that as often as you need to*

[P7, male]

Health Hub was viewed as a “one-stop shop” that contained relevant information regarding all aspects of their care, including appointments and results.

#### Concerns Regarding Digital Equity

Vulnerable patients and caregivers (including the older adults, those with lower levels of health or digital literacy, and those without access to an appropriate device) may struggle to use the system:

*Some older, really sick people might find this a bit hard to deal with...maybe their specialist would not put them into this program.*

[P3, female]

Allowing proxy or assisted completion might help address this.

Four themes related to the clinician-facing Epic EMR interface:

#### The ePRO System will Streamline the Process for Responding to Side Effects Between Clinic Visits

The ePRO system streamlined nursing workflows for responding to side effects between clinic visits by flagging severe side effects in real time, thus minimizing the need for ad hoc phone reviews:

*it gives you the ability to...get a quick summary of everyone...and what you need to do for the day.*

[N7, female]

Nurses appreciated that the responsibility for alerting and responding to severe side effects was shared between patients and clinicians. It also streamlined follow-up phone calls by providing preliminary data about the type and severity of side effects.

#### Excellent Alignment of ePRO System With Existing Epic Workflows

The ePRO system integrated well into existing clinician processes within Epic, ensuring key steps in the symptom monitoring workflow were not missed:

*I liked how there were multiple ways of doing things...like the treatment plan signing...no one forces you to enroll, but to consider it. If it wasn’t there, you’d forget about it.*

[O2, female]

Some aspects may require additional training, such as the use of Haiku (the mobile phone version of Epic; Epic Systems Corporation) for receiving severe side effect alerts, or the running of reports to update the cohort-level dashboard:

*We may not be using ‘reports’ as a usual thing, so that...may be a new step*

[N2, female]

#### The ePRO System Could Be Adapted to Each Clinician’s Personal Workflow

The ePRO system provided multiple ways of performing the same tasks, ensuring it aligned with each clinicians’s personal workflow:

*It was integrated pretty well into my normal workflow...it didn’t require me to go into any additional section to find exactly where it was.*

[O4, male]

This could be further improved by adapting the system for different clinician preferences (eg, alerts for severe side effects to both the Haiku app and the Epic EMR, routing of alerts to different nurse specialists).

#### Concerns Regarding Increased Workload Due to ePRO Symptom Monitoring

Reviewing results in real time was viewed as another clinical task to perform within an already resource-constrained environment. There were also some concerns regarding medico-legal liability if severe alerts were not responded to in a timely manner, particularly outside of business hours:

*You could see potentially medico-legally, someone reported a severe grade 3...you just signed off and you didn’t see it and then you will be legally liable, you didn’t respond to the symptoms.* [O9, male]

If a decision was made to turn the system off outside of business hours, participants suggested clearly stating this on Health Hub.

## Discussion

### Principal Findings

Usability and acceptance testing are critical in the development of digital health care tools, such as ePRO systems, that are fit for purpose and prioritize end-user satisfaction. Usability testing of medical devices is a regulatory requirement of the International Electrotechnical Commission (IEC 62366) [35] and the Food and Drug Administration [36]. However, there are no published examples of usability and acceptance testing of ePRO systems. To address this gap, we conducted formative, mixed methods usability and acceptance testing with end users prior to the implementation of a real-time ePRO symptom monitoring system for people receiving ICI.

We demonstrated high levels of prototype usability and acceptance on quantitative measures. Clinician usability was higher than patient and caregiver usability, whereas acceptance was similar across all participants. This may have reflected the clinicians’ higher levels of baseline digital literacy and their familiarity with the Epic EMR interface. In contrast, lower levels of patient and caregiver usability may have reflected lower baseline levels of digital literacy. While all patients and caregivers had prior access to Health Hub, only 2 out of 10 participants (20%) described themselves as “very confident” in using it. As the ePRO survey is contained within Health Hub, the usability of Health Hub more broadly will therefore affect the usability of the ePRO system. This highlights the challenge of embedding ePRO systems within existing EMR infrastructure, as the usability of the existing EMR infrastructure may limit the usability of the ePRO system. High levels of user acceptance were identified in the quantitative assessment across all participants.

This work builds upon the existing literature by demonstrating how ePRO system content and features can impact end-user usability and acceptance. Often, usability or acceptance issues are first detected during implementation and then addressed. However, this may affect end-users’ attitudes toward the ePRO system, which may be difficult to adjust following a negative user experience. This work, therefore, reinforces the importance of rigorously identifying and rectifying these issues prior to implementation, which may support sustained end-user engagement [37-39]. These findings are consistent with broader evidence from health information technology. Prior research on EMR usability has emphasized similar determinants of user acceptance, including perceived ease of use, workflow integration, and self-efficacy as key predictors of adoption [40,41]. Studies of clinical decision support systems also report that perceived usefulness, explainability, and interface design significantly affect user satisfaction and system uptake [42]. Similarly, work on patient portals demonstrates that usability and user experience depend heavily on perceived efficiency, emotional response, and ease of navigation [43,44]. By situating our ePRO results in this broader usability literature on health information technologies, we show that ePRO systems reflect many of the same human-computer interaction and acceptance challenges observed across digital health platforms. Finally, this work also highlights some of the challenges of optimizing the usability and acceptance of EMR-embedded ePRO systems as improvements may not always be possible to implement and should be considered at the point of system design.

Embedding the ePRO system within Epic had several advantages, including seamless workflow integration and real-time data capture, which was reflected in the participants’ qualitative feedback. However, we acknowledge that certain usability challenges, such as navigating within Health Hub and locating results within the Epic interface, may be specific to Epic’s menu structure, icon placement, and existing dashboard architecture and not generalizable to other EMRs. Other usability issues, such as the need for intuitive navigation to surveys, optimization of dashboard layout, management of real-time alerts, and digital equity and workload concerns, may reflect more general design and accessibility issues that may be relevant across ePRO platforms. While Epic’s widespread adoption in oncology will ensure the findings from this study are relevant, additional testing within non-Epic environments may be necessary to confirm the broader transferability of study findings.

This study has several strengths. First, testing followed best practice guidance outlined by Aiyegbusi [19]. This included assessing the broader IT system in which the ePRO system was nested and performing a combination of “on-site” testing to allow facilitators to observe how well participants interacted with the system and “off-site” testing where patients and caregivers tested in their own environment as this more closely resembled real-life use. Second, a broad range of end users participated, including people of different ages and levels of digital experience, providing varied perspectives on the system’s usability. Finally, testing was performed in real-time, enabling testing of both the ePRO system and the clinical workflow. This provided preliminary information regarding the workflow’s feasibility which will inform subsequent implementation.

Limitations of the testing included the low number of participants from certain subgroups (eg, culturally and linguistically diverse groups or people with disabilities). Future usability testing will need to be enriched for these subgroups. We performed predominantly formative testing. Summative data (such as time taken to complete tasks) were not collected. While this reflects the status of our ePRO system’s development (ie, co-design completed, but prototype yet to be finalized), this will need to be performed in future usability testing. Additional off-site testing for patients and caregivers could also be performed to increase the resemblance to how ePRO symptom monitoring would be conducted in real life. Finally, due to the lack of availability of IT staff, we were unable to make the suggested changes during the current testing period or perform iterative testing. Further testing will therefore be required once these changes have been implemented.

Future research will include additional postrevision usability and acceptance testing, with an emphasis on summative data not collected during the current round of testing. Participants from vulnerable groups, including older participants, those with a disability, and those from culturally and linguistically diverse backgrounds, will be specifically included in this round of testing to ensure the final ePRO system is broadly accessible. Additional strategies to improve digital equity may include structured onboarding (face-to-face demonstrations, step-by-step guides or optional training during routine appointments) to improve user confidence and engagement [45,46], allowing proxy-assisted completion by caregivers or with a clinician during clinic visits [45,47], and offering the interface in multiple languages [46]. This will be followed by implementation and assessment of the sustainability of the ePRO symptom monitoring system, including whether the efforts made upfront to assess usability result in higher levels of sustained end-user engagement.

### Conclusion

The ePRO system prototype demonstrated high levels of end-user usability and acceptance. Feedback will be incorporated into future ePRO prototype updates prior to its implementation into routine care. This study highlights the value in comprehensively assessing usability and acceptance prior to implementation to ensure the ePRO system’s content and functionality are fit for purpose. This will ensure the benefits of ePRO symptom monitoring are realized in routine cancer care.

## Supplementary material

10.2196/79694 Multimedia Appendix 1 Supplementary materials for the usability and acceptance testing study, including pretesting reading materials, the usability testing procedures and interview guide, and the customized Unified Theory of Acceptance and Use of Technology questionnaire.

## Abbreviations

CPQ-12: 12-item Computer Proficiency Questionnaire
EMR: electronic medical record
ePRO: electronic patient-reported outcome
ICI: immune checkpoint inhibitor
irAE: immune-related adverse event
MDPQ-16: 16-item Mobile Device Proficiency Questionnaire
REDCap: Research Electronic Data Capture
SUS: System Usability Scale
UTAUT: Unified Theory of Acceptance and Use of Technology
