# Supplementary material for: Usability and Acceptance Testing of an Electronic Patient-Reported Outcome Symptom Monitoring System for People Receiving Immune Checkpoint Inhibitors: Mixed Methods Study
Source: JMIR Form Res. 2026 Mar 18;10:e79694. doi: 10.2196/79694 (PMC12998712; doi:10.2196/79694)
Supplement: Multimedia Appendix 1 [file formative-v10-e79694-s001.docx]

SUPPLEMENTARY TEXT, TABLES AND FIGURES

Section 1: Usability testing pre-reading materials

Section 2: Usability testing procedures and interview guide

Section 3: Customised Unified Theory of Acceptance and Use of Technology (UTAUT) Questionnaire

Section 1: Usability testing pre-reading materials

Section 2: Usability testing procedures and interview guide

Usability and acceptance testing of an electronic patient-reported outcome (ePRO) symptom monitoring system for people receiving immunotherapy

TESTING PROCEDURES AND INTERVIEW GUIDE

| INTRODUCTION (10 minutes) |
| --- |

Welcome and introductions

- Thank you for making the time to take part in today’s study.
- My name is (insert name). I am a researcher at Peter MacCallum Cancer Centre. I will be guiding you through today’s testing session.
- Would you like to introduce yourself briefly to the other participants? Please tell us your name, whether you are a patient/ carer/ nurse/ doctor, and one sentence on why you decided to take part in the study.

Briefing session

- We are trying to understand what people think about a new app which allows people to report symptoms of their cancer treatment at any time. This system has been designed with patients, carers and clinicians at Peter Mac over the past 2 years. We would like to know how easy it is to use this system and what we can do to make it easier to use.
- There are 3 parts to today’s session:
  - We will first show you how to use the system
  - You will then be given a scenario in which you will be given a character to play. This will be either a patient receiving imunotherapy, the carer for someone receiving immunotherapy, a doctor or a nurse caring for a person recceiving immunotherapy.
  - The scenario will run as follows:
    - The doctor will enrol the person into ePRO symptom monitoring when starting their immunotherapy treatment
    - The patient/ carer will complete a survey about their symptoms (or those of the person they are caring for) on your choice of device (computer, mobile phone or tablet). Once you have completed the survey, you will be given some advice about what to do about your symptoms.
    - The nurse will then review the patient or carer’s responses on a dashboard. If there are any severe symptoms, they will receive a notification to a mobile phone.
    - The doctor will then review the patient or carer’s responses in Epic. You will be asked to interpret the patient’s responses and describe how you would discuss this with the patient. You will then unenrol the patient from ePRO symptom monitoring.
  - To make the scenario as realistic as possible, it will work in real-time. This means that whatever responses the patient/ carer enters will be what the nurse and doctor will see.
  - During the scenario, I will give you step-by-step instructions on what we want you to do. I will then ask you to perform the task while I watch and take notes. We will try not to help you as we want to see how easily you use the system. Just try your best! If can’t progress any further, we will of course help you so you can continue with the scenario.
  - Once you have finished the scenario, we will ask you some questions about how you found the tasks, what was easy/ difficult, and how we could improve the ePRO system.
- Housekeeping
  - Please listen to each other and respect differing opinions. Please avoid disruptions or talking over one another during the testing session. Anything said during today’s testing session should remain confidential.
  - If you need to stop and have a break at any time, please let me know. If you don’t want to continue with the session at any point, please let me know.
  - Please note that as this session is meant to test our system, technical glitches may occur. If this happens, we will do our best to rectify these quickly and continue the session.
  - The whole session should take 80 minutes to complete.
  - If you have any questions at any time during the session, please ask.
- Does anyone have any questions or would like anything clarified before we begin?

| TRAINING (10 minutes) + SCENARIO (40 minutes) |
| --- |

1. Training (10 mins)
2. Patient/ carer
   1. Select preferred device- computer or tablet/ mobile phone with Health Hub
   2. Start logged into Health Hub
   3. Trainer will demonstrate how to use welcome message, complete surveys, receive immediate advice about what to do about symptoms and how to interpret, review self-management advice related to their symptoms, review results and education library. The demonstration will follow the script below:

This is the Health Hub where you can find surveys to report your symptoms on immunotherapy treatment. You can see that you have two incomplete tasks in your ‘to do list’. Start with the welcome message. This message explains why we are doing ePRO symptom monitoring, how often you will complete the surveys (once a week + on demand), links to phone numbers for your nurse consultant at the hospital, links to your previous survey results and an education library. After viewing the welcome message, click ‘I understand’ to return to the home page and access the symptom survey.

The first page of the survey asks if you’ve had any changes to your health in the last 7 days. If you click ‘yes’, you will continue with the survey. If you click ‘no’, the survey ends here. This page appears only after completing the survey a set number of times.

You will then go through the survey and select any symptoms you’ve had in the last 7 days. Each page displays symptoms by body system. If you click on a symptom, you will be asked some other questions about it.

After you’ve completed the survey, there will be a summary page which shows what symptoms you’ve reported, how severe they are, and what you need to do immediately about them. For severe symptoms, you’ll be advised to contact the hospital immediately during business and outside of business hours. For moderate symptoms, you’ll be advised to contact the hospital within 24 hours during business hours. For mild symptoms, you do not need to tell the hospital. You can discuss these at your next clinic visit. You may also receive some advice about what you can do at home. You will see this once you close the survey.

You can review and edit your answers before submitting the survey. Once submitted, your responses will immediately be available in your medical record for your doctors and nurses to look at.

If you had any mild symptoms, a new task will be added to the ‘to-do list’. The new task will take you to an education library where you can read some information about what to do about your symptoms at home. The education library also has general information about immunotherapy, managing steroid side-effects, and handy phone numbers.

You can also look at your previous responses in the ‘trends dashboard’ which is located in the menu sidebar. Here, you can see how each symptom has changed over time. You will need to click ‘load more trends’ to view all the side-effect graphs, which are displayed in alphabetcal order by side-effect.

1. Nurse
   1. Device: computer with Epic + mobile phone with Haiku
   2. Start logged into Epic (computer) and Haiku (mobile phone)
   3. Trainer will demonstrate how to respond to severe alerts (push notification to Haiku, go to Epic and review patient’s ePRO results), how to respond to moderate alerts (my reports🡪 run report 🡪 how to interpret dashboard, how to access patient file and review ePRO results). The demonstration will follow the script below:

Once the patient submits their survey, this will be available immediately in Epic.

You can review these on a dashboard, which will show all the patients who are currently enrolled in ePRO monitoring that you are looking afer. To access the dashboard, click on ‘my reports’ 🡪 ‘PMC SEMS Care Companion Overview’. The dashboard shows all patients enrolled in ePRO symptom monitoring, the date of their last completed survey, and their survey results. Moderate symptoms are highlighted in yellow, and you will need to contact the patient within 24 hours during business hours to discuss them (if they don’t contact you before then). If you click on an individual patient, you can also see a summary of their responses as a graph.

If the patient reports a severe symptom, you will receive a notification via the Haiku app on your mobile phone. Click on the notification to view the survey results, where servere side-effects are highlighted in red. You’ll need to call the patient immediately to discuss symptom management. The patient will also be asked to call the hospital immediately to discuss their symptoms.

1. Doctor
   1. Device: computer with Epic
   2. Start logged into Epic
   3. Trainer will demonstrate how to enrol and unenrol patients, how to see if a patient is enrolled, how to access and interpret ePRO results. The demonstration will follow the script below:

To enrol the patient in ePRO symtpom monitoring, sign the first cycle on immunotherapy treatment plan. A ‘best practice advisory’ will pop-up to add ePRO symtom monitoring to the care plan. The patient will then complete the survey to report their side-effects.

To look at your patient’s ePRO responses, go to ‘chart review’. A banner with a link to the results section will appear at the top of the page. You can also find this under ‘Visit and wrap up’ 🡪 ‘SEMS summary’. Click on ‘go to results’ to see their ePRO responses as a graph. You can also see this information as a table next to the graph. Moderate and severe side-effects are highlighted in yellow and red, respectively.

To unenrol the patient, go to the ‘Visit and wrap up’. Where it says ‘would you like to delete SEMS’, click on ‘remove’. This will automatically stop surveys from being sent to the patient.

1. Reset computers post training- unenrol patient from ePRO symptom monitoring and delete questionnaire series.
2. Scenario (40 mins)

- We will now begin the scenario.
- Each of you has a piece of paper describing the character that you will play during the testing session as well as the tasks you will be asked to perform.
- Please take a moment to read this now.
- During the scenario, I will give you step-by-step instructions on what we want you to do. I will then ask you to perform the task while I watch and take notes. We will try not to help you as we want to see how easily you use the system. Just try your best! If can’t progress any further, we will of course help you so you can continue with the scenario. As we go through the system, if you find something that you think needs to be clarified or changed, please tell us and we will write it down (Table 1).
- Does anyone have any questions or would like anything clarified before we begin?

1. Doctor
   1. Start scenario with doctor logged onto Epic with Chris Ehaq’s medical record open, clinic encounter opened, on ‘Visit and wrap up’ tab.
   2. Instructions to participant:
      1. You are a doctor about to start our patient Chris on immunotherapy (ipilimumab and nivolumab). They have agred to participate in ePRO symptom monitoring.
      2. Please sign the next cycle of the ‘ipilimumab and nivolumab’ treatment plan and add ePRO symptom monitoring to the treatment plan
2. Patient/ Carer
   1. Start scenario with patient/ carer logged onto Health Hub as Chris Ehaq
   2. Instructions to participant:
      1. You are a patient (Chris)/ carer for a patient (Chris) who is on immunotherapy (ipilimumab and nivolumab) for stage IV melanoma. You are about to have your 2^nd^ treatment next week. You have just received a message asking you to complete a survey before your appointment. You log into Health Hub to complete your survey. In the past week, you have had the following symptoms: moderate rash, mild itch, severe diarrhoea
      2. Please review the welcome message, then return to the survey.
      3. Please report your symptoms by completing the survey.
      4. (once reaches screen with immediate advice)- please read this information and tell us what symptoms you have reported, how severe they are, and what you would do about each of the symptoms (who/ when/ how you could contact)
      5. Please have a look at the advice for what to do about your itch and rash at home.
      6. Please have a look at the graphs for your symptoms over time. Can you please describe what you see? How has your rash/ diarrhoea/ itch changed over time? It is getting better, worse or staying the same?
3. Nurse
   1. Start scenario with nurse logged into Haiku and Epic. Within Epic, have Chris Ehaq’s medical record open to ‘Chart review’.
   2. Instructions to participant:
      1. You are a clinical nurse consultant who will be reviewing alerts for moderate and severe symptoms for people with melanoma receiving immunotherapy. One of your patients is Chris.
      2. Moderate symptoms: please open the dashboard to review Chris’ symptoms. Please explain what you see on the dashboard. What symptoms has he/she experienced? How severe are they? What do you plan to do about them?
      3. Severe symptoms: as Chris has reported a severe symptom, you have also received a notification via Haiku to your mobile phone. Open this notification in the app. Which symptom(s) were severe? What would you do about it?
4. Doctor
   1. Doctor should still be logged onto Epic with Chris Ehaq’s medical record open, clinic encounter opened, on ‘Visit and wrap up’ tab.
   2. Instructions to participant:
      1. You are now seeing our patient Chris in clinic for a pre-immunotherapy treatment review.
      2. Please open the patient’s ePRO results within the EMR.
      3. Please review their symptoms. What symptoms have been reported? How severe are the symptoms?
      4. Chris has now decided that they want to stop ePRO symptom monitoring. Please show us how you would unenrol them from the system.

| INTERVIEW (30 minutes) |
| --- |

These are sample questions for our semi-structured interviews that will be refined further throughout the project. Commensurate with the semi-structured interview method, the questions will also evolve during each interview conversation based on participants’ responses.

1. On a scale of 1 to 10, how satisfied are you with the ePRO symptom monitoring system overall?
2. How easy was it to navigate and use the system?
3. What did you like or dislike about the system?
4. How would you compare your experience today with any of your previous experiences reporting side-effects between visits to hospital?
5. Do you think this system could work in the real-world? Why or why not? If not, how would you improve the system to make it work in the real-world?
6. Do you foresee any risks to patients/ clinicians if this system was used in the real world? (e.g. safety, quality of patient care)

| INTERVIEW CLOSE |
| --- |

Do you have any other questions or comments before we end the session?

Thank you again for your time today. Iris will send you out a voucher to thank you for your time. Once the testing is finished, we look forward to sharing the results with you.

Iris will also send you an email with links to two surveys that you can complete in your own time after today’s session. Please try to complete these within the next day or so, so you don’t forget how today’s session went!

Section 3: Customised Unified Theory of Acceptance and Use of Technology (UTAUT) Questionnaire

Patient:

|  | Strongly Disagree | Disagree | Neither | Agree | Strongly Agree |
| --- | --- | --- | --- | --- | --- |
| 1. I found the system useful for recording my side-effects | 1 | 2 | 3 | 4 | 5 |
| 1. If I use the system, it will make it easier for me to communicate with the hospital | 1 | 2 | 3 | 4 | 5 |
| 1. I found the presentation of the system clear and understandable. | 1 | 2 | 3 | 4 | 5 |
| 1. I found the content of the system clear and understandable | 1 | 2 | 3 | 4 | 5 |
| 1. Learning to use the system was easy. | 1 | 2 | 3 | 4 | 5 |
| 1. Overall, I found the system easy to use. | 1 | 2 | 3 | 4 | 5 |
| 1. People who influence my behaviour (eg. family, friends, my doctors and nurses) think I should use the system | 1 | 2 | 3 | 4 | 5 |
| 1. People whose opinion I value (eg. family, friends, my doctors and nurses) would like me to use the system | 1 | 2 | 3 | 4 | 5 |
| 1. In general, I support the use of the system. | 1 | 2 | 3 | 4 | 5 |
| 1. I have the resources needed to use the system (eg. a smartphone or computer, internet access, family support). | 1 | 2 | 3 | 4 | 5 |
| 1. I have the knowledge necessary to use the system (eg. basic computer skills). | 1 | 2 | 3 | 4 | 5 |
| 1. The system fits well with my lifestyle | 1 | 2 | 3 | 4 | 5 |
| 1. The system is compatible with other technology I use day to day | 1 | 2 | 3 | 4 | 5 |
| 1. I can get help from others if I have difficulty using the system (eg. from family, hospital staff) | 1 | 2 | 3 | 4 | 5 |
| 1. If made available to me, I would use the system in the next 3 months | 1 | 2 | 3 | 4 | 5 |

Carer:

|  | Strongly Disagree | Disagree | Neither | Agree | Strongly Agree |
| --- | --- | --- | --- | --- | --- |
| 1. I found the system useful for recording side-effects for the person I care for | 1 | 2 | 3 | 4 | 5 |
| 1. If I use the system to report side-effects, it will make it easier for us to communicate with the hospital | 1 | 2 | 3 | 4 | 5 |
| 1. I found the presentation of the system clear and understandable. | 1 | 2 | 3 | 4 | 5 |
| 1. I found the content of the system clear and understandable. | 1 | 2 | 3 | 4 | 5 |
| 1. Learning to use the system was easy. | 1 | 2 | 3 | 4 | 5 |
| 1. Overall, I found the system easy to use. | 1 | 2 | 3 | 4 | 5 |
| 1. People who influence my behaviour (eg. the person I care for, family, friends, my doctors and nurses) think I should use the system | 1 | 2 | 3 | 4 | 5 |
| 1. People whose opinion I value (eg. the person I care for, family, friends, my doctors and nurses) would like me to use the system | 1 | 2 | 3 | 4 | 5 |
| 1. In general, I support the use of the system. | 1 | 2 | 3 | 4 | 5 |
| 1. I have the resources needed to use the system (eg. a smartphone or computer, internet access, family support). | 1 | 2 | 3 | 4 | 5 |
| 1. I have the knowledge necessary to use the system (eg. basic computer skills). | 1 | 2 | 3 | 4 | 5 |
| 1. The system fits well with my lifestyle | 1 | 2 | 3 | 4 | 5 |
| 1. The system is compatible with other technology I use day to day | 1 | 2 | 3 | 4 | 5 |
| 1. I can get help from others if I have difficulty using the system (eg. from family, hospital staff) | 1 | 2 | 3 | 4 | 5 |
| 1. If made available to me, I would use the system in the next 3 months | 1 | 2 | 3 | 4 | 5 |

Clinician:

|  | Strongly Disagree | Disagree | Neither | Agree | Strongly Agree |
| --- | --- | --- | --- | --- | --- |
| 1. I found the system useful for reviewing my patients’ side-effects | 1 | 2 | 3 | 4 | 5 |
| 1. If my patients use the system, it will improve my patient’s ability to communicate with the hospital | 1 | 2 | 3 | 4 | 5 |
| 1. I found the presentation of the system clear and understandable | 1 | 2 | 3 | 4 | 5 |
| 1. I found the content of the system clear and understandable | 1 | 2 | 3 | 4 | 5 |
| 1. Learning to use the system was easy. | 1 | 2 | 3 | 4 | 5 |
| 1. Overall, I found the system easy to use. | 1 | 2 | 3 | 4 | 5 |
| 1. People who influence my behaviour (eg.my patients, my colleagues, senior management) think I should use the system | 1 | 2 | 3 | 4 | 5 |
| 1. People whose opinion I value (eg. my colleagues, senior management) would like me to use the system | 1 | 2 | 3 | 4 | 5 |
| 1. In general, I support the use of the system. | 1 | 2 | 3 | 4 | 5 |
| 1. I have the resources needed to use the system (eg. access to a computer, time to review side-effects) | 1 | 2 | 3 | 4 | 5 |
| 1. I have the knowledge necessary to use the system (eg. basic computer skills). | 1 | 2 | 3 | 4 | 5 |
| 1. The system fits well with my day-to-day work | 1 | 2 | 3 | 4 | 5 |
| 1. The system is compatible with other technology I use day-to-day | 1 | 2 | 3 | 4 | 5 |
| 1. I can get help from others if I have difficulty using the system (eg. my colleagues, IT) | 1 | 2 | 3 | 4 | 5 |
| 1. If made available to me, I would use the system in the next 3 months | 1 | 2 | 3 | 4 | 5 |
